# Extensive neurological recovery from a complete spinal cord injury: a case report and hypothesis on the role of cortical plasticity

**DOI:** 10.3389/fnhum.2013.00290

**Published:** 2013-06-25

**Authors:** Ann S. Choe, Visar Belegu, Shoko Yoshida, Suresh Joel, Cristina L. Sadowsky, Seth A. Smith, Peter C. M. van Zijl, James J. Pekar, John W. McDonald

**Affiliations:** ^1^Department of Neurology, Johns Hopkins University School of MedicineBaltimore, MD, USA; ^2^International Center for Spinal Cord Injury, Hugo W. Moser Research Institute at Kennedy Krieger, Inc.Baltimore, MD, USA; ^3^F. M. Kirby Research Center for Functional Brain Imaging, Kennedy Krieger InstituteBaltimore, MD, USA; ^4^Russell H. Morgan Department of Radiology and Radiological Science, Johns Hopkins University School of MedicineBaltimore, MD, USA; ^5^Physical Medicine and Rehabilitation, Johns Hopkins University School of MedicineBaltimore, MD, USA; ^6^Department of Radiology and Radiological Science, Institute of Imaging Science, Vanderbilt UniversityNashville, TN, USA

**Keywords:** spinal cord injury, trauma, diffusion tensor imaging, magnetization transfer imaging, resting state fMRI, plasticity

## Abstract

Neurological recovery in patients with severe spinal cord injury (SCI) is extremely rare. We have identified a patient with chronic cervical traumatic SCI, who suffered a complete loss of motor and sensory function below the injury for 6 weeks after the injury, but experienced a progressive neurological recovery that continued for 17 years. The extent of the patient's recovery from the severe trauma-induced paralysis is rare and remarkable. A detailed study of this patient using diffusion tensor imaging (DTI), magnetization transfer imaging (MTI), and resting state fMRI (rs-fMRI) revealed structural and functional changes in the central nervous system that may be associated with the neurological recovery. Sixty-two percent cervical cord white matter atrophy was observed. DTI-derived quantities, more sensitive to axons, demonstrated focal changes, while MTI-derived quantity, more sensitive to myelin, showed a diffuse change. No significant cortical structural changes were observed, while rs-fMRI revealed increased brain functional connectivity between sensorimotor and visual networks. The study provides comprehensive description of the structural and functional changes in the patient using advanced MR imaging technique. This multimodal MR imaging study also shows the potential of rs-fMRI to measure the extent of cortical plasticity.

## Case presentation

The patient, a 58-year-old male, endured vertebral fractures at cervical levels C3–C6 in a motor vehicle accident at the age of 22. The patient did not have any respiratory problem and his stay in the ICU was limited to 5 days. Following the injury, the patient underwent manual motor testing of lower and upper body muscles groups as well as sensory testing. The motor testing involved examination of flexion and extension of upper body (shoulder, elbow, and wrist), and lower body joints (hip, knee, and ankle), and sensory testing involved examination of sensation to pinprick and light touch on both hands and feet. Initially, the patient had no motor or sensory function below the level of injury. Motor and sensory function at the sacral level was also absent, which was tested during the bladder evacuation and catheter placement procedure. This injury is equivalent to an ASIA A (ASIA: American Spinal Injury Association), or a “complete” injury, according to today's ASIA impairment scale (AIS) (Maynard et al., 1997), which did not exist at the time of the patient's injury.

Spinal cord stabilization was accomplished with a halo. His rehabilitation regimen consisted of active-assisted range of motion (ROM) exercises of the lower and upper limb performed at home. Lower limb ROM exercise was performed for 30 min, three times a day, and the upper limb ROM exercise was performed for 30 min, two times a day. The exercise was performed at home, assisted by his family members.

**Figure 1:**
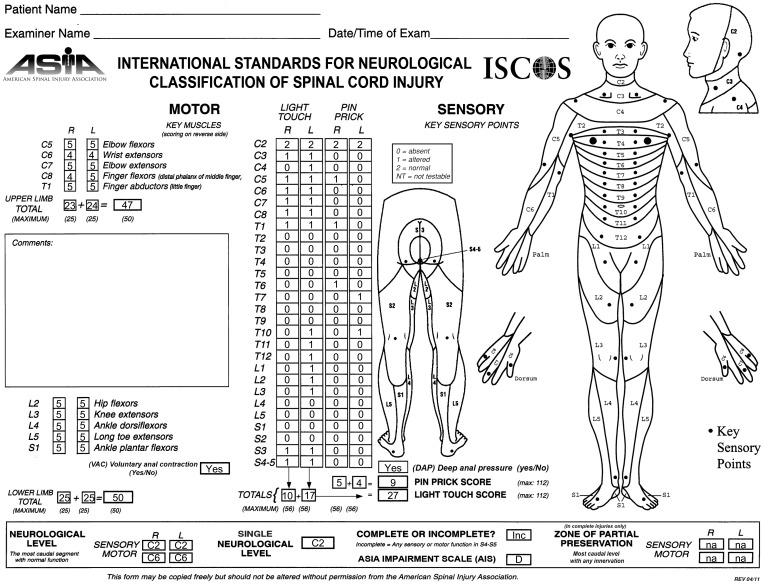
**ASIA classification of the patient after recovery, performed according to the International Standards for Neurological Classification**.

He experienced slow and progressive neurological recovery that continued for 17 years after the injury. The first neurological recovery, movement of the left big toe (a grade 1 motor function), occurred 6 weeks after the injury. The first recovery of sensation occurred 6 months after the injury in the form of a painful dysesthetic sensation in the pelvis. Eleven months after the injury, the patient was able to perform complex upper body motor functions such as sitting up without assistance. Complex lower body motor functions such as ability to walk unassisted were recovered 15 months after the injury. Concomitantly, the patient recovered autonomic nervous system functions (i.e., a high degree of bladder and bowel functions were recovered up to 64 months after the injury) although many remain abnormal (Table 1). Currently, his injury is categorized as ASIA D. His latest AIS evaluation (Figure 1) showed that he has regained 94% of motor function in the upper body and 100% in the lower body. The patient manifests incomplete and asymmetric motor recovery in his hands—i.e*.*, his left hand is more functional then the right one. He has also regained 23% of sensory function in the upper body (above the level of T7), but only 10% in the lower body. The patient reports that his walking and functions he performs with his feet depend heavily on visual feedback; he also manifests autonomic dysreflexia. It should be noted that the AIS evaluation of the motor function is based on muscle strength in the extremities, and that sensory function is evaluated based on conscious sensation of light touch and pin prick on the skin, and does not include proprioception.

**Table 1:** **Detailed timeline of the patient's recovery**.

	**Date**	**Description**
0	Sep-74	Accident
1	Nov-74	Flicker of contraction present at left big toe. No sensory recovery
2	Mar-75	Flicker of contraction present at left quadriceps
3	Mar-75	Dysesthetic (abnormal) sensation at pelvis
4	Mar-75	Flick of sensation present at left bicep
5	Mar-75	Flick of sensation present at left fingers
6	Apr-75	Flicker of contraction present at right big toe
7	May-75	Begin to gain trunk movement
8	May-75	Begin working on trunk balance
9	Jun-75	Sit with 50% assistance
10	Jun-75	Flicker of contraction present at left triceps
11	Oct-75	Sit without assistance
12	Dec-75	Active movement of left arm against gravity
13	Jan-76	Stand with 50% assistance
14	Feb-76	Regain ability to grip with hands
15	May-76	Regained ability to ejaculate
16	Jun-76	Stand without assistance
17	Sep-76	First swim
18	Jan-77	Begin to gain control of bladder movement (incomplete)
19	Jan-78	First walk 40 feet without assistance
20	Jun-78	Could walk in community
21	Jan-79	Begin to gain control of bowel movements (incomplete)
22	1978–1980	Functional recovery rate begins to plateau
23	Apr-91	First run

The 1974 motor accident [0] resulted in a complete loss of motor and sensory function below the injury epicenter that lasted 6 weeks after the injury.

Initial regaining of motor and sensory function [1, 3] was then followed by a long but steady recovery process that continued for 17 years after the injury, where at the end of the 17 years, he was able to run without assistance [23].

## Background

The spinal cord is a conduit for the exchange of information between the brain and the body, and damage to it disrupts conduction of sensory and motor signals across the lesion epicenter (Belegu et al., 2007). The extent of the neurological impairment following spinal cord injury (SCI) varies, and the severity of the injury limits the subsequent neurological recovery (McDonald and Sadowsky, 2002; Kirshblum et al., 2004). Severely injured SCI patients, classified as ASIA A on the AIS (Maynard et al., 1997), experience limited neurological recovery that often does not translate to functional improvements. Patients with less severe injuries, classified as ASIA B-D, experience greater recovery (Kirshblum et al., 2004). This is further corroborated by a retrospective study of 173 traumatic SCI patients with 5-year follow up (Vazquez et al., 2008), which reported relatively good recovery results for patients with incomplete injury; at the time of their discharge, 33.3% the ASIA B patients, 76.4% of the ASIA C patients, and 100% of the ASIA D patients showed some degree of neurological recovery. The same study also reported, however, that 94% of the ASIA A patients remained complete upon discharge without any neurological recovery and none of them were functional at the time of their discharge (Vazquez et al., 2008). Chances of neurological recovery for ASIA A patients become even smaller in chronic phase of SCI (McDonald and Sadowsky, 2002).

Two variables that contribute to the capacity for neurological recovery are well understood. First, preservation of longitudinally oriented axonal tracts in the white matter is required (Blight, 1983; Nathan, 1994). Second, proper conductance through the spared white matter axons and hence proper myelination is required. However, only 5% of intact axons in the white matter tracts are needed to procure locomotion following SCI in cats (Blight, 1983), while in humans, locomotion can recover after resection of 25% of the spinal cord (Nathan, 1994), indicating that preservations of axons and myelination are not proportional to the degree of neurological recovery. Task-activated functional MRI (fMRI) studies show different activation pattern between patients who experience neurological recovery and those who do not (Jurkiewicz et al., 2007, 2010), suggesting that cortical plasticity may be a third variable. While the occurrence of cortical plasticity subsequent to SCI has been observed (Endo et al., 2007; Jurkiewicz et al., 2007, 2010), it is unclear how it leads to neurological recovery.

Here, we present a report of a patient with a chronic cervical traumatic SCI, who suffered a complete loss of motor and sensory function below the injury epicenter for 6 weeks after the injury, and experienced a slow and progressive neurological recovery that continued for 17 years. The extent of the recovery is so remarkable that it represents a pragmatic cure from trauma induced complete paralysis. We utilized advanced MRI technologies such as diffusion tensor imaging (DTI), magnetization transfer imaging (MTI), and resting state fMRI (rs-fMRI) to investigate the patient's late recovery. While DTI and MTI showed abnormalities in the spinal cord that indicate axonal and myelin damage, but are not associated with the patient's late recovery, rs-fMRI provided a picture of cortical plasticity that is associated with the patient's late recovery.

## Materials and methods

### Participants

Spinal cord DTI, brain DTI, and brain rs-fMRI data of healthy controls were obtained from previously acquired and separate databases that exist in the International Center for Spinal Cord Injury (ICSCI) and F.M. Kirby center. Spinal cord imaging was performed in ten healthy volunteers (21–49 years, mean 33, M/F ratio: 6/4). For brain imaging, DTI was performed in six age matched healthy volunteers (55–61 years, mean 59, M/F ratio: 2:4), and rs-fMRI was performed in 21 (22–61 years, mean 32, M/F ratio: 11/10; available on web: www.nitrc.org/projects/multimodal) healthy volunteers (Landman et al., 2011). Only the six healthy participants from the brain DTI database were age matched due to the limited number of older healthy participants in the spinal cord DTI and brain rs-fMRI databases. All participants gave their informed consent, and the study protocol was approved by the institutional review board at Johns Hopkins University.

### Image acquisition

**Figure 2:**
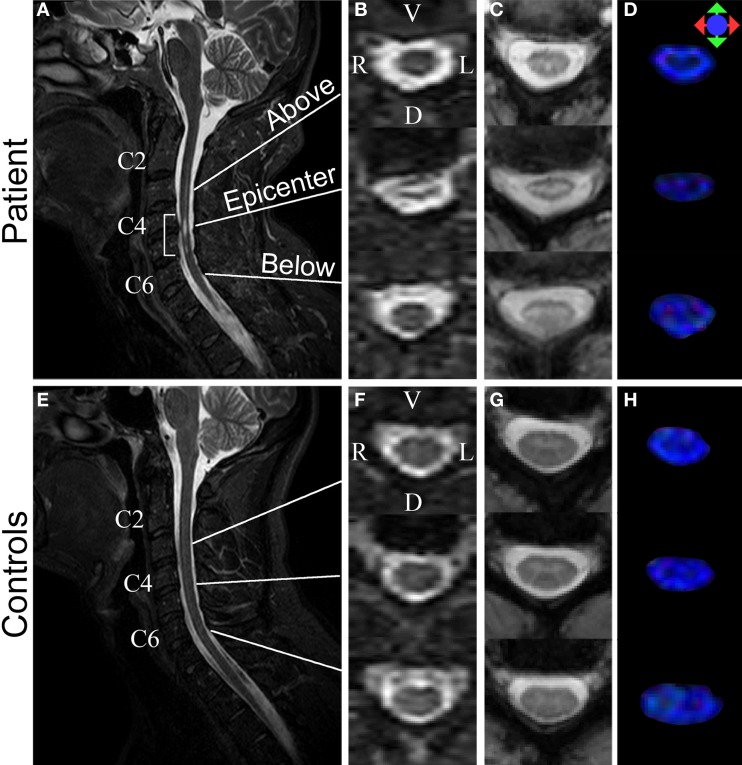
**Structural MR images acquired from the patient (top row) and a control (bottom row)**. **(A,E)** Sagittal T2-w images. Bracket in **(A)** highlights injury epicenter of the patient. **(B,F)** Axial T2-w images. **(C,G)** Axial MTCSF images. **(D,H)** Axial FA color maps. The FA maps are color-coded using a standard diffusion color-encoding scheme (r/l: red, a/p: green, s/i: blue). Each row of **(B–D)** shows a section from: (1) above the injury epicenter, (2) injury epicenter, and (3) below the injury epicenter. Images from the corresponding cervical levels in the control are shown in each row of **(F–H)**.

Participants were scanned on a Philips 3T scanner. The scan room was lit during the scan, but the lights were kept off inside the scanner bore. For spinal cord imaging, DTI was performed using a multi-slice, pulsed gradient spin echo (PGSE) sequence (*b* = 0, 500 s/mm^2^, 16 diffusion weighting directions, TR/TE = 6300/63 ms, SENSE factor = 2, 1.5 × 1.5 × 3 mm^3^ original resolution (axial sections of 3 mm thickness), 0.57 × 0.57 × 3 mm^3^ final resolution (after zero-fill), scan time = 2 min, 16-channel neurovascular coil) (Figures 2D,H). MT-weighted images were acquired using a three dimensional (3D) spoiled gradient-echo sequence with an EPI readout (EPI factor = 3, TR/TE = 102/13 ms, α = 9°, and SENSE factor = 2, 0.61 × 0.69 × 3 mm^3^ resolution, scan time = 8 min, 16-channel neurovascular coil) (Figures 2C,G). A sagittal T2 weighted (T2-w) was also acquired (Figures 2A,B,E,F). The total scan time for the spinal cord imaging was 28 min.

Brain DTI was performed using a multi-slice PGSE sequence (*b* = 0, 700 s/mm^2^, 32 directions, TR/TE = 7270/71 ms, SENSE factor = 2.5, 2.2 × 2.2 × 2.2 mm^3^ original resolution, 0.83 × 0.83 × 2.2 mm^3^ final resolution, scan time = 5 min, 16-channel neurovascular coil). A high resolution T1 weighted (T1-w) MPRAGE image (TR/TE = 10/6 ms, SENSE factor = 2, 1.10 × 1.10 × 1.17 mm^3^ original resolution, 0.83 × 0.83 × 1.17 mm^3^ final resolution, scan time = 5 min, 16-channel neurovascular coil) was also obtained.

Each rs-fMRI scan of the controls (Landman et al., 2011) was acquired using a single-shot SENSE-EPI (TR/TE = 2000/30 ms, SENSE factor = 2, 3 × 3 × 3 mm^3^ resolution, 1 mm gap, scan time = 420 s, 32-channel head coil). Identical imaging parameters were used to acquire the rs-fMRI data of the SCI patient, with these differences: (1) time of each run: 360 s, (2) number of runs acquired: 8. For the controls, the first 360 s of the data was used for further analysis. Total scan time for the brain imaging was 30 min. The rs-fMRI scans were always acquired after the brain T1-w image acquisition to allow the participants to get acclimated to the noise and the new environment inside the scanner. The participants were instructed to stay as still as possible with their eyes closed during the entire scan. A dataset from one of the healthy controls was excluded due to excessive motion.

### Image registration for spinal cord imaging

Basic eddy current correction was performed on the spinal cord and brain DTI datasets using CATNAP (Coregistration, Adjustment, and Tensor-solving, a Nicely Automated Program) (Landman et al., 2007), which performs volume-wise coregistration using a method based on FLIRT (FMRIB's Linear Imaging Registration Tool, Oxford, UK) (Jenkinson et al., 2002). Each diffusion weighted image was registered to an initial *b* = 0 s/mm^2^ (b0, non-diffusion weighted image) volume using a six degrees of freedom, rigid-body registration. Diffusion gradient tables were updated to account for any rotation.

The registration of the MTI dataset to the mean b0 volume was performed in two steps. First, the MTI dataset was registered to the b0 volume using a 3D, six degrees of freedom, rigid body transformation using a similar method proposed by Maes et al. (1999). This 3D registration procedure was then followed by a two-dimensional (2D) registration, using a three degrees of freedom rigid body transformation that involved two in-plane translations and one rotation. The second coregistration procedure was implemented for added accuracy of the result (Smith et al., 2010).

### Measurement of whole cord and white matter cross-sectional area

The whole cord and white matter atrophy of the patient's spinal cord was measured using fractional anisotropy (FA) maps. We first separated the spinal cord and the surrounding cerebrospinal fluid (CSF) area from the rest of the neck region by performing manual segmentation. This was done by placing ellipsoidal binary masks around the spinal cord and the surrounding CSF area. Once the segmentation of the spinal cord and CSF was done, the whole cord cross-sectional area (wca) of each slice of the controls and the patient was measured automatically using the Otsu's thresholding method, therefore reducing rater bias. The segmented FA maps of the cords were then thresholded at 0.2—the same threshold used during the subsequent fiber tractograophy for the column-specific data analysis—to create the masks of the white matter only area (wma). We have previously shown that FA measures performed in this manner have acceptable inter-rater and test-retest reliability (Smith et al., 2010), and this is expected to extend to the volume measures based on this parameter.

### Diffusion fiber tractography of spinal columns and creation of column profiles

After the registration of DTI and MTI images and the calculation of DTI- and MTI-derived quantities, diffusion fiber tractography was performed using DTIStudio (Jiang et al., 2006). Higher contrast MTCSF (MT-weighted signal intensity relative to cerebrospinal fluid) images—compared to T2-w images and FA maps—were used to manually draw regions of interest (ROIs) within left and right lateral, dorsal, and ventral columns of the spinal cords. This manual ROI selection was performed on every third section along the entire cervical cord, to be used as seed regions for the fiber tractography to follow. Note that the registered MTCSF images are in the same image space as that of the DTI images. FA threshold of 0.2 and a maximum tract turning angle of 60° were used as the fiber tractography stopping criterion. Spurious fibers, such as those that cross from one spinal column to the next or move out of the spinal cord, were manually excluded.

Once each spinal column was reconstructed, vertebrae level of C2 and C6 were identified and the corresponding column profiles (Smith et al., 2010; Cohen-Adad et al., 2013) were created. In our previous study (Smith et al., 2010), typical neck length of study participants was found to be 75 mm, which corresponds to 25, 3 mm thickness image sections. Using this information, each column profile was spatially normalized to 25 points spanning C2–C6.

### Injury region specific data analysis

Tables 2, 3 report the column profile values of the patient (mean) and the controls (mean ± SD) for three spinal cord regions: (1) above the injury epicenter, (2) injury epicenter, and (3) below the injury epicenter. The injury epicenter was identified using the patient's T2-w images (Figure 2B). One vertebrae level (approximately 15 mm) above the beginning of the lesion site was defined as the region “above” the injury epicenter, and one vertebrae level down from the end of the lesion site was defined as the “below” the injury epicenter. The three spinal cord regions of the controls were defined to match the location of the patient's regions.

**Table 2:**
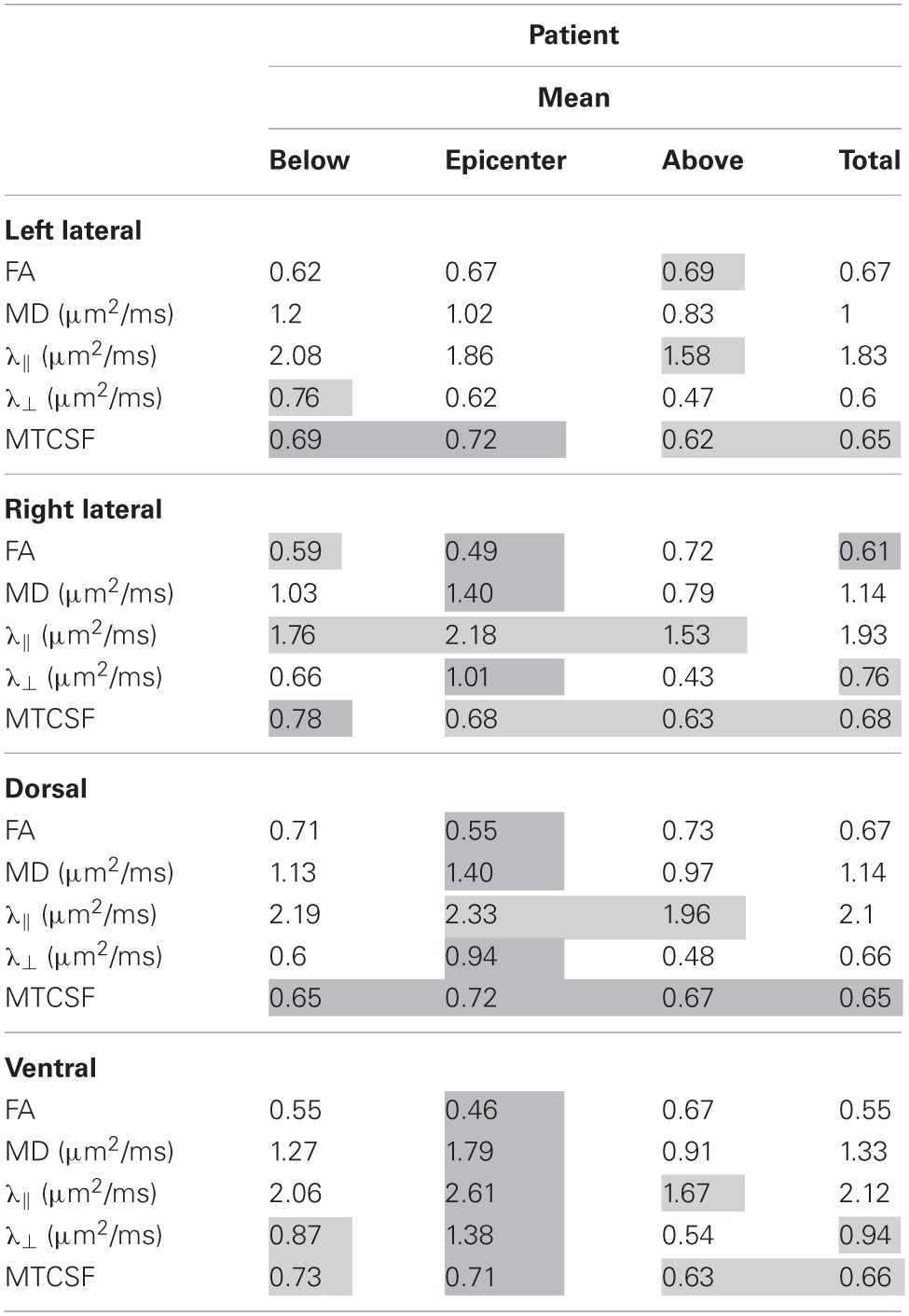
**Injury region specific data comparison of the patient**.

Imaging parameters (FA, MD, λ_ǁ_, λ_⊥_, and MTCSF) were measured for the regions of above the injury, injury epicenter, and below the injury.

Values reported are mean of each injury region. Examining the cervical cord in an injury region specific way provides better insight into the actual changes occurring along the patient's cervical spinal cord, as opposed to looking at a single average values of an entire spinal column.

Difference compared to the controls.

Within normal range

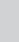
*Difference is bigger than 1 × SD*

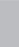
*Difference is bigger than 2 × SD*

**Table 3:** **Injury region specific data comparison of controls**.

	**Controls**			
	**Mean ± SD**			
	**Below**	**Epicenter**	**Above**	**Total**
**Left lateral**				
FA	0.68 ± 0.10	0.74 ± 0.10	0.79 ± 0.09	0.75 ± 0.10
MD (μm^2^/ms)	0.99 ± 0.16	1.00 ± 0.24	0.93 ± 0.25	0.97 ± 0.22
λ_ǁ_ (μm^2^/ms)	1.89 ± 0.23	2.01 ± 0.34	1.98 ± 0.34	1.98 ± 0.29
λ _⊥_ (μm^2^/ms)	0.54 ± 0.18	0.51 ± 0.23	0.42 ± 0.21	0.47 ± 0.22
MTCSF	0.48 ± 0.08	0.48 ± 0.11	0.48 ± 0.09	0.49 ± 0.10
**Right lateral**				
FA	0.74 ± 0.09	0.79 ± 0.07	0.78 ± 0.09	0.78 ± 0.08
MD (μm^2^/ms)	0.98 ± 0.18	0.89 ± 0.16	0.94 ± 0.23	0.94 ± 0.21
λ_ǁ_ (μm^2^/ms)	1.99 ± 0.22	1.90 ± 0.26	1.97 ± 0.32	1.98 ± 0.30
λ_⊥_ (μm^2^/ms)	0.48 ± 0.19	0.39 ± 0.13	0.43 ± 0.21	0.43 ± 0.19
MTCSF	0.48 ± 0.10	0.48 ± 0.10	0.48 ± 0.08	0.49 ± 0.10
**Dorsal**				
FA	0.70 ± 0.14	0.78 ± 0.09	0.77 ± 0.08	0.76 ± 0.10
MD (μm^2^/ms)	1.11 ± 0.31	0.98 ± 0.17	1.02 ± 0.15	1.04 ± 0.22
λ _ǁ_ (μm^2^/ms)	2.14 ± 0.36	2.10 ± 0.22	2.17 ± 0.20	2.16 ± 0.28
λ _⊥_ (μm^2^/ms)	0.59 ± 0.32	0.42 ± 0.17	0.45 ± 0.16	0.49 ± 0.22
MTCSF	0.47 ± 0.08	0.48 ± 0.10	0.46 ± 0.09	0.47 ± 0.09
**Ventral**				
FA	0.59 ± 0.07	0.61 ± 0.07	0.66 ± 0.08	0.61 ± 0.08
MD (μm^2^/ms)	1.10 ± 0.17	1.15 ± 0.25	1.09 ± 0.20	1.16 ± 0.25
λ _ǁ_ (μm^2^/ms)	1.92 ± 0.28	2.03 ± 0.36	2.03 ± 0.29	2.05 ± 0.35
λ _⊥_ (μm^2^/ms)	0.69 ± 0.14	0.71 ± 0.22	0.62 ± 0.18	0.71 ± 0.22
MTCSF	0.52 ± 0.11	0.52 ± 0.08	0.51 ± 0.09	0.54 ± 0.11

Imaging parameters (FA, MD, λ_ǁ_, λ_⊥_, and MTCSF) were measured for cervical spinal cord regions that correspond with the subject's above the injury, injury epicenter, and below the injury region of the subject. Values reported are mean ± SD of each injury region.

Each cell in Table 2 is color coded according to the degree of difference between the values of the controls and patient: (1) cells without color indicate difference less than one SD, (2) light gray cells indicate difference of more than one SD but less than twice the SD, (3) dark gray cells indicate difference of bigger than twice the SD. When DTI and MTI derived parameters were averaged along the whole cord, only FA of the right lateral column and MTCSF of the dorsal column were found to have changes of more than twice the SD (Table 2). A detailed look at the Tables 2, 3, however, reveals that there are substantial changes of DTI- and MTI-derived parameters above, at, and below the injury epicenter of different spinal columns. FA, MD, and λ_⊥_ altered more than two SD at the injury epicenter of right lateral, dorsal, and ventral columns. λ_ǁ_ altered more than two SD at the injury epicenter of ventral column. MTCSF changed more than two SD at the injury epicenter of the left lateral, dorsal, and ventral column, below the injury epicenter at the right lateral, dorsal, and ventral column, and above the injury epicenter at the dorsal column. All DTI-derived quantities values for the left lateral column were within the normal range, suggesting the left lateral column is the most well preserved spinal column. This corresponds with the patient's clinical presentation, who presents higher motor score for the left side of the body than the right.

### Brain DTI data analysis

The structural integrity of the patient's brain was investigated through T1-w imaging and DTI, using an atlas-based analysis (ABA) method (Faria et al., 2010, 2011). The diffusion weighted images were first co-registered to one of the b0 images using a 12-mode affine transformation of Automated Image Registration (AIR) (Woods et al., 1998). The six elements of the diffusion tensor, FA, and mean diffusivity (MD) were calculated. After skull-stripping, the images were first normalized to the JHU-DTI-MNI “Eve” template (Jiang et al., 2006) with a nine-parameter affine transformation of AIR using b0 image. Then, a non-linear transformation using dual-contrast large deformation diffeomorphic metric mapping (LDDMM) (Beg et al., 2005) was applied, using FA and MD images. These procedures are reciprocal, so the inverse-transformed brain parcellation map was superimposed onto the original MRI images, and led to parcellation of the brain into 130 anatomical structures (Oishi et al., 2008, 2011). Some of the structures were then further segmented into gray and white matter using an FA threshold of 0.25 in each subject, followed by minimum manual adjustment. This resulted in 46 additional parcellations, yielding a total of 176 regions. When the quantitative values were obtained, the cerebrospinal fluid spaces were excluded by an MD threshold at 0.0020 mm^2^/s. The final results of the anatomical quantification included volume, FA and MD for each subject. The non-linear image transformation and the atlas-based parcellation were performed using DiffeoMap and RoiEditor (http://www.MriStudio.org) (Oishi et al., 2008).

### Brain resting state fMRI preprocessing and analysis

The preprocessing of rs-fMRI data was performed using SPM8 (www.fil.ion.ucl.ac.uk/spm) (Friston et al., 1994) and Matlab (Natick, MA). The preprocessing pipeline involved: (1) slice timing correction, (2) motion correction, (3) co-registration, (4) unified segmentation-normalization (Ashburner and Friston, 2005), (5) high pass filtering with 0.005 Hz cutoff, and (6) a spatial smoothing using 6 mm full-width at half-maximum Gaussian kernel. Finally, principal component analysis was performed, followed by group independent component analysis (GICA) (Calhoun et al., 2001) of all runs from the healthy controls and the subject using the GIFT software (http://mialab.mrn.org/software/gift). An order selection using minimum description length (MDL) criterion (Li et al., 2007) was performed and a total of 39 independent components were initially estimated. After GICA, three were eliminated due to low similarity measures obtained using ICASSO toolbox; (Himberg et al., 2004) 24 were rejected due to artifacts (e.g., motion) and physiology (e.g., cardiac pulsation).

For each remaining IC, the magnitude of temporal fluctuations was computed to provide a measure of within-network connectivity (WNC). This was done by first converting the back reconstructed time courses to percent signal change measurements for normalization purpose, and computing the root mean square (RMS) of the normalized time course values. The between network connectivity (BNC) was computed for every pair of functional networks as well, as the Pearson's correlation of the network time courses.

## Results

### MRI-based structural evaluation of a chronically injured spinal cord

In the T2-weighted MR images, a lesion is visible in C4–C5 of the injured spinal cord (Figures 2A,B), location of which agrees with the known location of the injury epicenter. No lesion is observed in a healthy spinal cord (Figures 2E,F).

**Figure 3:**
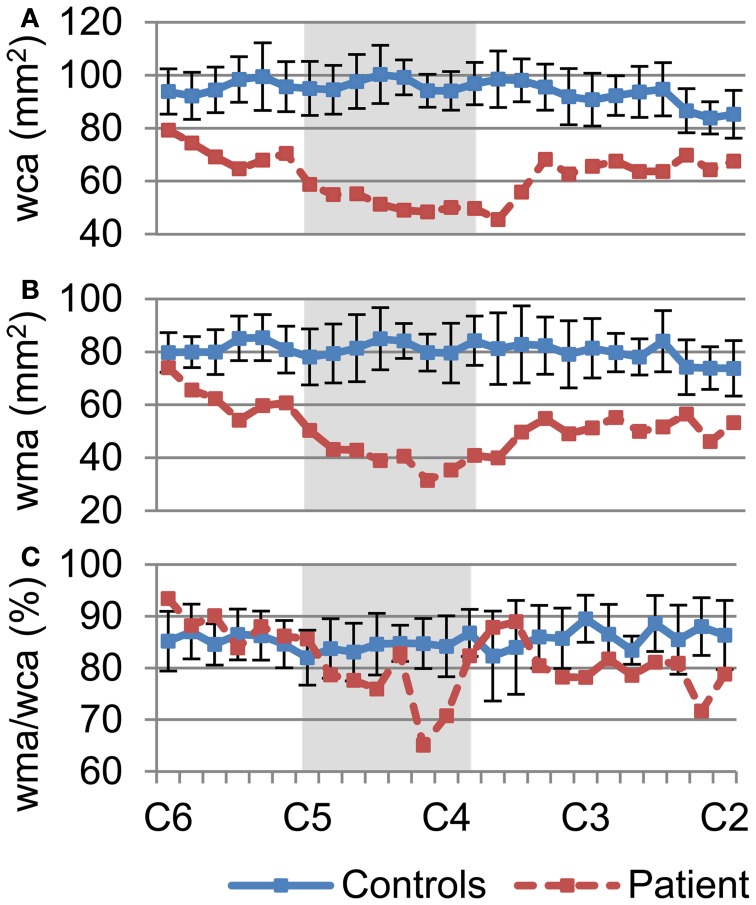
**Quantification of whole cord and white matter atrophy, as a measure of white matter tissue sparing throughout the cervical spinal cord. (A)** Degree of whole cord atrophy, as measured by the whole cord area, **(B)** white matter area, and **(C)** the ratio of wma over wca, was measured between C2–C6. Values of the controls (blue line) are mean ± SD. Plot of wca **(A)** and wma **(B)** demonstrate atrophy of the patient's spinal cord not only at the injury epicenter (dark area) but throughout the cervical cord. Plot of ratio of wma over wca **(C)** shows marked decrease of white matter around the injury epicenter.

The degree of preservation of longitudinally oriented axonal tracts in white matter was investigated by assessing the whole cord and white matter atrophy of the patient's spinal cord. We measured wca (Figure 3A) and wma (Figure 3B) of each slice in the cervical cord for the controls (mean ± SD; SD: standard deviation; blue line) and the patient (mean; red line) using FA maps (Figures 2D,H). Gray boxes in each profile represent the injury epicenter. The wca and wma are reduced throughout the cervical cord (more than one SD decrease) and maximally reduced in the injury epicenter (55 and 62%, respectively). These profiles show that atrophy spans the entire measured area within the cervical cord but is more pronounced in the injury epicenter. In addition, a measure of the area of spared white matter tissue was provided by the wma to wca ratio (Figure 3C). A sharp decrease of the ratio (23%) shows that the focal atrophy at the epicenter of the injury is due to white matter atrophy.

**Figure 4:**
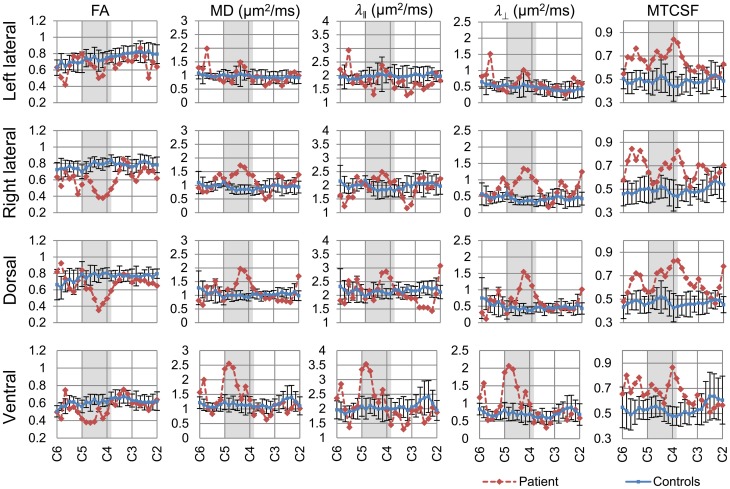
**Column-specific data comparison of DTI- and MTI-derived quantities of the controls (blue line) and the patient (red line)**. FA, MD, λ_ǁ_, and λ_⊥_, and MTCSF values of each spinal column were measured between C2–C6. The resulting column profiles are shown. Darker area within the plots indicates the patient's injury epicenter. Values reported for the controls are mean ± SD. FA value decreased, while MD, λ_ǁ_, and λ_⊥_ increased at the injury epicenter. MTCSF value increased throughout the cervical cord.

We aimed to assess the axonal integrity and myelination within major spinal cord columns (dorsal, lateral, and ventral columns) of the spared white matter tissue, where dorsal columns convey sensory function while the lateral and ventral columns convey primarily motor functions. Segmentation of each of the columns was performed using DTI fiber tractography, and column profiles for each of the DTI- and MTI-derived quantities were created (Figure 4). Quantities derived from DTI [FA, MD, axial diffusivity (λ_ǁ_), and radial diffusivity (λ_⊥_)] and MTI (MTCSF) have been previously shown to be sensitive to axonal and myelin integrity (Beaulieu, 2002; Song et al., 2002, 2005; Smith et al., 2005; Farrell et al., 2010; Landman et al., 2010; Levesque et al., 2010; Choe et al., 2012), with DTI-derived quantities more sensitive to axon and MTI-derived quantities more sensitive to myelin. Next, we used MTCSF images to place ROIs for DTI tractography. MTCSF increased by more than one SD at the injury epicenter in all spinal columns, and its changes were diffuse and extended throughout the cervical cord. Unlike MTCSF, FA decreased by more than one SD primarily at the injury epicenter along all spinal columns, with the greatest decrease observed in the dorsal column. The decrease in FA was more pronounced in the right lateral column than in the left lateral column (Figure 4). These observations are consistent with the patient's neurological manifestation, as the patient's sensory deficit is worse than motor deficit and motor deficit on the right side of the body is worse than left side of the body. MD, λ_ǁ_, and λ_⊥_increased focally at the injury epicenter. However, the pattern of changes of these quantities is different from that of FA, with changes being more pronounced in the right versus left lateral column and the ventral column being the most pronounced (Figure 4, Tables 2, 3).

### MRI-based evaluation of the brain in a chronic spinal cord injury patient

#### Structural imaging

Considering the extensive structural changes we observed in the spinal cord of the patient, we wanted to assess if such changes extend to the brain. The structural integrity of the patient's brain was investigated through T1-w imaging and DTI, using an atlas based whole brain analysis with 176 parcellations. Structure volume, FA, and MD measurements of appropriate controls and the patient were compared using a Mann-Whitney U-test. No significant differences between the controls and the patient were observed (Table 4). The lack of alterations of these quantities indicates the SCI has not produced significant structural changes in the patient's brain.

**Table 4:** **Comparison of the DTI-derived quantities (structure volume, FA, and MD) between the controls and the patient using Mann–Whitney U test**.

	***p*-value**				***p*-value**				***p*-value**				***p*-value**		
**Column ID**	**Volume**	**FA**	**Trace**	**Column ID**	**Volume**	**FA**	**Trace**	**Column ID**	**Volume**	**FA**	**Trace**	**Column ID**	**Volume**	**FA**	**Trace**
SPG_L	0.286	0.857	0.829	SS_L	0.571	1.000	0.757	SFG_R	0.857	1.000	0.808	UNC_R	0.571	0.571	0.471
CingG_L	0.857	0.857	0.654	EC_L	0.857	0.571	0.894	MFG_R	0.857	1.000	0.706	PCT_R	0.857	0.571	0.557
SFG_L	0.286	1.000	0.745	UNC_L	0.571	0.857	0.687	IFG_R	0.286	1.000	0.463	MCP_R	0.857	0.286	0.753
MFG_L	0.857	1.000	0.680	PCT_L	0.286	0.857	0.690	PrCG_R	1.000	1.000	0.518	Fx_R	0.286	0.286	0.094
IFG_L	0.571	1.000	0.697	MCP_L	1.000	1.000	0.768	PoCG_R	1.000	0.857	0.550	GCC_R	0.286	0.286	0.329
PrCG_L	1.000	1.000	0.673	Fx_L	0.286	0.571	0.785	AG_R	0.857	0.857	0.458	BCC_R	0.857	0.857	0.310
PoCG_L	0.286	0.857	0.640	GCC_L	0.286	0.571	0.321	PrCu_R	0.571	0.857	0.712	SCC_R	0.286	0.571	0.702
AG_L	0.286	0.571	0.875	BCC_L	0.857	0.571	0.220	Cu_R	1.000	1.000	0.680	RLIC_R	1.000	0.857	0.991
PrCu_L	1.000	1.000	0.581	SCC_L	0.286	0.286	0.938	LG_R	0.857	0.857	0.943	RedNc_R	0.857	0.286	0.969
Cu_L	0.857	1.000	0.773	RLIC_L	0.857	0.571	0.585	FuG_R	0.571	1.000	0.501	Snigra_R	0.286	0.857	0.692
LG_L	1.000	0.571	0.958	RedNc_L	1.000	0.857	0.973	PHG_R	0.571	1.000	0.818	TAP_R	0.857	0.857	0.496
Fu_L	0.857	1.000	0.817	Snigra_R	0.286	0.571	0.987	SOG_R	0.286	0.857	0.957	Caud_R	1.000	0.857	0.657
PHG_L	0.571	0.857	0.974	TAP_L	0.857	0.286	0.225	IOG_R	0.571	0.857	0.541	Put_R	0.286	0.571	0.271
SOG_L	1.000	1.000	0.677	Caud_L	0.286	0.571	0.764	MOG_R	0.857	0.857	0.777	Thal_R	0.286	0.571	0.889
IOG_L	0.286	0.857	0.881	Put_L	0.857	0.857	0.591	ENT_R	0.286	0.857	0.911	GP_R	0.857	0.857	0.283
MOG_L	0.857	0.857	0.760	Thal_L	0.286	0.571	0.911	STG_R	0.571	1.000	0.480	Midbrain_R	0.857	0.571	0.607
ENT_L	0.286	0.571	0.779	GP_L	0.286	0.857	0.560	ITG_R	0.571	0.857	0.537	Pons_R	0.857	1.000	0.750
STG_L	0.857	1.000	0.794	Midbrain_L	0.857	0.286	0.492	MTG_R	0.286	1.000	0.431	Medulla_R	1.000	0.286	0.836
ITG_L	0.857	0.571	0.947	Pons_L	0.571	0.857	0.798	LFOG_R	0.857	0.571	0.408	SPwm_R	0.667	1.000	0.983
MTG_L	0.286	1.000	0.796	Medulla_L	0.571	0.286	0.746	MFOG_R	0.857	1.000	0.482	Cingwm_R	0.667	1.000	0.896
LFOG_L	0.571	0.571	0.763	SPWM_L	0.667	0.667	0.984	SMG_R	0.571	0.857	0.551	SFWM_R	0.667	0.667	0.936
MFOG_L	0.286	1.000	0.611	Cingwm	0.333	1.000	0.948	RG_R	0.571	1.000	0.479	MFWM_R	1.000	0.667	0.871
SMG_L	0.571	0.857	0.877	SFWM_L	0.333	0.667	0.990	Ins_R	0.286	1.000	0.282	IFWM_R	0.333	0.667	0.502
RG_L	0.571	0.857	0.532	MFWM_L	0.667	0.667	0.958	Amyg_R	0.857	0.571	0.622	PrCWM_R	1.000	1.000	0.682
Ins_L	0.286	0.857	0.385	IFWM_L	1.000	0.667	0.855	Hippo_R	0.571	0.571	0.912	PoCWM_R	0.667	0.667	0.842
Amyg_L	0.571	0.857	0.765	PrCWM_L	1.000	0.667	0.794	cerebellum_R	1.000	0.286	0.627	AWM_R	0.333	0.667	0.909
Hippo_L	0.571	0.286	0.859	PoCWM_L	0.333	0.667	0.848	CST_R	0.857	0.286	0.324	PreCuWM_R	0.667	0.667	0.911
cerebrellum_L	0.571	0.857	0.805	AWM_L	0.333	1.000	0.915	ICP_R	1.000	0.286	0.858	CuWM_R	0.667	1.000	0.482
CST_L	0.571	0.286	0.534	PrCuWM_L	1.000	0.667	0.892	ML_R	0.286	0.286	0.673	LWM_R	0.667	0.333	0.642
ICP_L	1.000	0.571	0.994	CuWM_L	0.333	1.000	0.629	SCP_R	0.286	0.286	0.621	Fuwm_R	0.667	0.667	0.893
ML_L	0.286	0.571	0.810	LWM_L	0.667	1.000	0.767	CP_R	0.286	0.286	0.279	SOWM_R	0.667	1.000	0.867
SCP_L	0.571	0.571	0.655	Fu_WM_L	1.000	0.667	0.926	ALIC_R	0.571	0.286	0.301	IOWM_R	1.000	1.000	0.490
CP_L	0.286	0.286	0.144	SOWM_L	0.667	1.000	0.823	PLIC_R	0.286	0.286	0.448	MOWM_R	0.667	1.000	0.739
ALIC_L	0.286	0.286	0.449	IOWM_L	0.667	0.667	0.890	PTR_R	0.286	0.857	0.914	STWM_R	0.667	1.000	0.705
PLIC_L	0.286	0.286	0.633	MOWM_L	1.000	0.667	0.987	ACR_R	0.286	0.571	0.958	ITWM_R	0.333	1.000	0.800
PTR_L	0.286	1.000	0.650	STwm_L	0.667	0.667	0.994	SCR_R	0.571	0.571	0.646	MTWM_R	0.667	1.000	0.671
ACR_L	0.286	0.571	0.948	ITWM_L	0.333	0.667	0.856	PCR_R	0.286	0.857	0.900	LFOWM_R	0.333	1.000	0.681
SCR_L	0.857	0.857	0.737	MTWM_L	0.333	0.667	0.927	CGC_R	1.000	0.857	0.921	MFOWM_R	0.333	0.667	0.801
PCR_L	0.286	0.286	0.997	LFOWM_L	1.000	1.000	0.732	CGH_R	0.571	0.571	0.929	SMWM_R	0.333	0.667	0.483
CGC_L	0.857	0.571	0.980	MFOWM_L	0.333	0.667	0.888	Fx/ST_R	0.571	0.571	0.570	RGWM_R	0.333	0.667	0.953
CGH_L	0.857	0.857	0.877	SMWM_L	0.333	0.667	0.709	SLF_R	0.286	0.857	0.725	cerebrellumwm_R	0.333	0.667	0.587
Fx/ST_L	0.857	0.286	0.952	RGWM_L	0.333	0.667	0.737	SFO_R	1.000	0.571	0.712	IFO_L	0.286	0.571	0.750
SLF_L	0.286	0.857	0.972	cerebrellumwm_L	0.667	0.667	0.632	IFO_R	0.286	0.857	0.399	CingG_R	0.857	1.000	0.706
SFO_L	1.000	0.571	0.936	SPG_R	0.571	1.000	0.769	SS_R	0.571	0.857	0.966	EC_R	0.857	0.571	0.608

SPG, superior parietal lobule; CingG, cingulate gyrus; SFG, superior frontal gyrus; MFG, middle frontal gyrus; IFG, inferior frontal gyrus; PrCG, precentral gyrus; PoCG, postcentral gyrus; AG, angular gyrus; PrCu, pre-cuneus; Cu, cuneus; LG, lingual gyrus; Fu, fusiform gyrus; PHG, parahippocampal gyrus; SOG, superior occipital gyrus; IOG, inferior occipital gyrus; MOG, middle occipital gyrus; ENT, entorhinal area; STG, superior temporal gyrus; ITG, inferior temporal gyrus; MTG, middle temporal gyrus; LFOG, lateral fronto-orbital gyrus; MFOG, middle fronto-orbital gyrus; RG, gyrus rectus; Ins, insular; Amyg, amygdala; Hippo, hippocampus; cerebrellum, cerebellum; CST, corticospinal tract; ICP, inferior cerebellar peduncle; ML, medial lemniscus; SCP, superior cerebellar peduncle; CP, cerebral peduncle; ALIC, anterior limb of internal capsule; PLIC, posterior limb of internal capsule; PTR, posterior thalamic radiation; ACR, anterior corona radiata; SCR, superior corona radiata; PCR, posterior corona radiata; CGC, cingulum (cingulate gyrus); CGH, cingulum (hippocampus); Fx/ST, fornix (cres)/stria terminalis; SLF, superior longitudinal fasciculus; SFO, superior fronto-occipital fasciculus; SMG, supramarginal gyrus; IFO, inferior fronto-occipital fasciculus; SS, sagittal stratum; EC, external capsule; UNC, uncinate fasciculus; PCT, pontine crossing tract; MCP, middle cerebellar peduncle; Fx, fornix; GCC, genu of corpus callosum; BCC, body of corpus callosum; SCC, splenium of corpus callosum; RLIC, retrolenticular part of internal capsule; RedNc, red nucleus; Snigra_R, substancia nigra; TAP, tapatum; Caud, caudate nucleus; Put, putamen; Thal, thalamus; GP, globus pallidus; Midbrain, midbrain; Pons, pons; Medulla, medulla; SPWM, superior parietalwm; Cingwm, cingulum wm; SFWM, superior frontal wm; IFWM, inferior frontalwm; MFWM, middle frontalwm; PrCWM, precentralwm; PoCWM, postcentralwm; AWM, angularwm; PrCuWM, pre-cuneuswm; CuWM, cuneus wm; LWM, lingual wm; Fu_WM, fusiform wm; SOWM, superior occipital wm; IOWM, inferior occipital wm; MOWM, middle occipital wm; STwm, superior temporalwm; ITWM, inferior temporalwm; MTWM, middle temporalwm; LFOWM, lateral fronto-orbitalwm; MFOWM, middle fronto-orbitalwm; SMWM, supramarginalwm; RGWM, rectuswm; cerebrellumwm, cerebellum wm.

#### Resting state fMRI (rs-fMRI)

**Figure 5:**
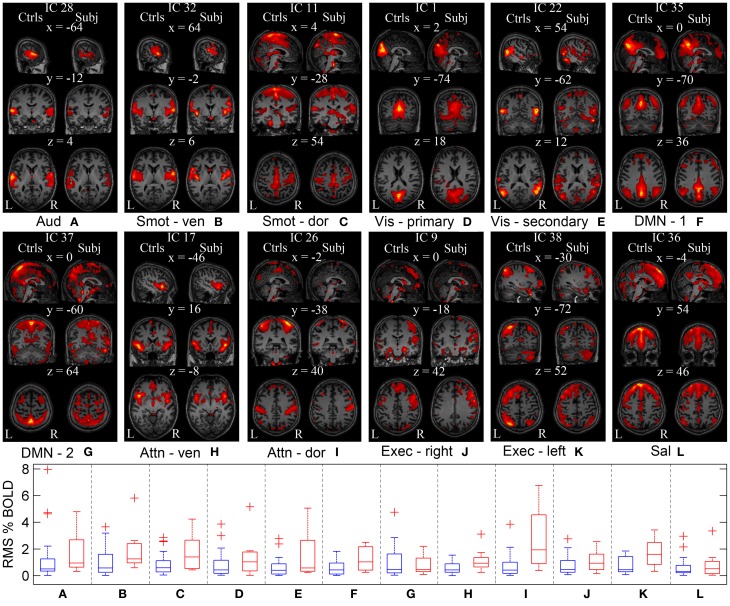
**Functional networks of the controls and the patient estimated using GICA**. GICA was performed to delineate same functional networks in the controls and the patient. ICs are overlaid on the Montreal Neurological Institute (MNI) template. **(A–L)** First column corresponds to the sagittal, coronal, and axial view of the controls' functional networks. Second column corresponds to the patient's functional networks. Coordinates (in mm) for each view are indicated on top of the subfigures, along with the IC numbers assigned during GICA. WNC of the controls (in blue) and the patient (in red) for each network are shown as a series of box plots. On each box, central mark is the median and edges of the box are the 25th and 75th percentiles. Aud, auditory; Smot, seonsorimotor; Vis, visual; DMN, default mode network; Attn, attention; Exec, executive; Sal, salience.

In order to investigate cortical plasticity, we used rs-fMRI, which measures spontaneous fluctuations in BOLD (blood oxygenation level dependent) signal in the absence of externally-cued task. This allows for direct comparison with controls regardless of a patient's motor and sensory deficits. Functional networks were identified using GICA, which uses higher-order statistics to recover independent sources from multivariate data (Calhoun et al., 2001). It separates rs-fMRI data into components reporting on spontaneous fluctuations in intrinsic brain networks, and components reporting on nuisances such as head motion and cardiac pulsations. The procedure yielded 12 independent components (IC) representing different functional networks (Figure 5).

Within-network connectivity: For each IC, the magnitude of temporal fluctuations—the root mean square (RMS) of the component time course—provides a measure of within-network connectivity. The RMS % BOLD changes for each IC of appropriate controls and the patient are shown as series of box plots in Figure 5. The outliers in the plot were determined using a variable *w* (maximum whisker length), which was set to 1.5. Points were drawn as outliers if they are larger than q3 + *w*(q3 − q1) or smaller than q1 − *w*(q3 − q1), where q1 and q3 are the 25th and 75th percentiles, respectively. The plotted whisker extends to the adjacent value, which is the most extreme data value that is not an outlier. The median temporal modulations of the controls' ICs are between 0.3 and 0.5% of original BOLD MRI signal. The median temporal modulations of the patient's ICs are between 0.4 and 1.4% of average BOLD MRI signal, except for the dorsal attention and left executive networks with higher temporal modulation at 1.6 and 1.9%, respectively. The range of temporal modulations of the patient's ICs were bigger for auditory, dorsal somatosensory, secondary visual, and dorsal attention networks, compared to those of the controls. In general, magnitude of the network temporal modulation of the patient was bigger than that of the controls.

**Figure 6:**
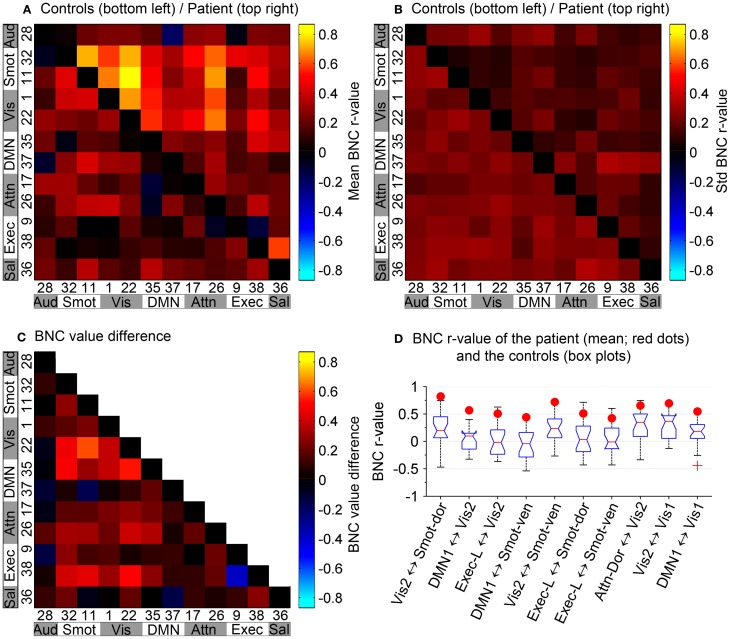
**Between-network connectivity. (A)** BNC correlation matrix shows synchrony between pairs of functional networks. **(B)** Combined matrix of the standard deviation of the corresponding BNC measurements. **(A,B)** In reference to the top–left to bottom–right diagonal axis, bottom–left portion corresponds to the controls and top–right portion corresponds to the patient. Diagonal elements have been zeroed for display purposes. **(C)** Difference between the controls and the patient's BNC measurements. **(D)** Box plots of controls' BNC measurements, with the patient's mean BNC values indicated by red dots. Shown are the 10 functional networks with the largest differences between the patient and controls.

Between-network connectivity: Synchrony between network time courses is referred to as between-network connectivity (BNC) (Joel et al., 2011). Figures 6A,B show a combined correlation matrix of the controls and the patient, and a combined matrix of the standard deviation of the corresponding BNC measurements, respectively. Figure 6C shows the difference between the controls and the patient's BNC measurements. Figure 6D shows box plots of the controls' BNC measurements, with the mean BNC value of corresponding functional networks of the patient indicated using red dots. Ten functional networks with the biggest mean BNC value difference between the controls and the patient are shown. Compared to controls, five functional networks of the patient, namely the sensorimotor, visual, DMN, attention, and executive networks, show increased synchrony with other networks. A decrease of functional connectivity is also observed between the left and right executive networks. The visual networks show a marked increase of connectivity with other functional networks such as the DMN, with the largest increase in the connectivity being observed between sensorimotor and visual networks. The patient has not regained sensory function in the lower body and relies heavily on vision to guide his lower body motor tasks. The increased connectivity between the sensorimotor and the visual networks is consistent with this clinical presentation and suggests compensatory functional changes in the patient's brain.

## Discussion

We used advanced MRI technologies to understand how a patient with a complete SCI achieved a pragmatic cure, defined as sufficient recovery of motor, sensory, and autonomic function to enable the patient to live and function independently. The patient achieved the recovery despite a significant atrophy, a focal FA decrease, and a diffuse increase of MTCSF in the residual white matter. This indicates that the patient's residual spinal cord tissue has sufficient integrity to enable function. The rs-fMRI showed increased functional connectivity between the patient's sensorimotor and the visual networks, indicating cortical plasticity; this cortical plasticity was not accompanied by structural changes in the brain.

### MRI-based structural evaluation of a chronically injured spinal cord

Whole cord cross-sectional area was shown to correlate with the motor and sensory scores of SCI patients (Lundell et al., 2011). However, the atrophy of the whole cord is accompanied by increased scar tissues in the cord and the degree of white matter atrophy can be greater than that of the whole cord, as our data shows (Figure 3). We used FA images, which can differentiate white matter from gray matter and scar tissues, to segment white matter from the injured cord. The wca and wma values of the healthy spinal cords measured using this approach were comparable to those values measured using higher resolution structural images acquired through 7T MRI (Sigmund et al., 2011), CT (Fountas et al., 1998), cadaver anatomy (Ko et al., 2004), as well as other previous histological methods (Kameyama et al., 1996; White et al., 1997; Gilmore et al., 2005). Our results showed that the patient's neurological recovery is achieved despite a 62% atrophy of the white mater tissue at the injury epicenter.

Structural integrity of the major columns directly affects neurological recovery of specific motor and sensory functions. Column-specific data analysis (Smith et al., 2010) enabled incorporation of manual and tractography-based ROI selection approach, allowing more time efficient data analysis, and comparison of DTI- and MTI-derived quantities of the controls and the patient in the residual tissue from the major columns (Figure 4). DTI and MTI are advanced MR imaging modalities that are sensitive to tissue microstructures. DTI provides an indirect measure of tissue structure on a microscopic scale by probing water diffusion (Basser et al., 1994), while MTI does so by measuring the magnetization interaction between bulk water protons and semi-solid macromolecular protons (Wolff and Balaban, 1989; Smith et al., 2005). FA, λ_ǁ_, and MTCSF are sensitive to changes in both axonal and myelin integrity, but previous studies have shown that λ_ǁ_ is more sensitive to axonal damages (Song et al., 2002, 2005; Budde et al., 2009) while FA is not specific (Farrell et al., 2010; Landman et al., 2010). In our patient, focal changes of DTI-derived quantities (Figure 4) were found that indicate mixed damage types over the cervical cord—i.e*.*, the reduction in FA is caused mainly by increases in λ_ǁ_ that could be due to axonal and myelin damage. MTCSF is more sensitive to myelination and the diffuse increase of MTCSF throughout the cervical cord (Figure 4) suggests diffuse demyelination or dysmyelination throughout the cervical cord. Overall, the DTI parameters describe the extensive damage but not how it affected the recovery.

A high correlation between FA and total AIS scores in chronic SCI patients has been described (Cohen-Adad et al., 2011). From that data we extrapolate a FA value (0.47) for chronic SCI patients with the same AIS score as our patient (124). The FA value of the patient (0.63) is higher than the extrapolated FA value (0.47). The higher FA of the patient is driven by the higher λ_ǁ_. The higher λ _ǁ_ value can be explained in part by the previously reported trend of MD and λ_ǁ_ change in different stages of traumatic injuries, with initial decrease during the acute phase of injury (Loy et al., 2007) and increased values during the chronic phase of injury (Naismith et al., 2009; Klawiter et al., 2011). This trend alone, however, does not fully explain the higher λ_ǁ_ observed in the patient, as the patient cohort observed in Cohen-Adad et al. (2011) are also chronic SCI patients with comparable delay after injury as the patient in this report. The higher λ_ǁ_ of the patient therefore may suggest higher axonal integrity. The DTI- and MTI-derived quantities of the controls were comparable to those reported in similar studies (Zackowski et al., 2009; Smith et al., 2010).

The small size of a spinal cord presents major imaging challenges. High resolution images are often desired, and the size makes the images more vulnerable to partial volume effect and various motions artifacts. While longer scan time and gating may be used to alleviate these issues to an extent, keeping patients with movement difficulties in the scanner for a long time is undesirable. Issue of misregistration cannot be ignored either, as the consequence of misregistration is greater in the spinal cord due to its size. One source of such misregistration is the use of an EPI read out during DTI acquisition, which can result in substantial eddy current effects. In order to minimize the effect of eddy current and misregistration, a robust registration scheme utilizing CATNAP (Farrell et al., 2007; Landman et al., 2007) was used to correct artifacts in DTI, using optimized input parameters (Farrell et al., 2008). While a more sophisticated eddy current correction scheme that uses field maps may improve the registration accuracy further, the tract-specific data comparison of this study showed good correlation between structural and functional information provided by MRI and clinical evaluation of the patient.

### MRI-based evaluation of the brain in a chronic spinal cord injury patient

Considering the extensive structural changes of the patient's spinal cord, and based on previous studies that report marked atrophy in the sensorimotor cortex (Crawley et al., 2004; Jurkiewicz et al., 2006; Wrigley et al., 2009; Freund et al., 2011; Henderson et al., 2011), our initial hypothesis was that we would observe similar gray matter atrophy in the patient's brain. Our result indicates, however, that the injury did not lead to significant structural changes in the patient's brain. One possible explanation is that unlike other SCI patients, the patient's structural changes were small and intracortical, and we did not have enough sample size and statistical power to detect the subtle changes. One study (Henderson et al., 2011), for example, shows that an extensive functional reorganization such as the one the patient demonstrates, may work to minimize the anatomical atrophy in the sensorimotor cortex. This hypothesis is also in part supported by the initial observations of chronic deafferentiation and its effect on cortical anatomy (Florence et al., 1998) where major functional reorganization following limb amputation of a macaque monkey was accompanied by significant increase of intracoritcal connections but not by any significant changes of major thalamocortical fiber tracts.

In order to differentiate functional networks, GICA (Calhoun et al., 2001) was performed using all eight runs of the subject and one run from 20 healthy controls. This enabled us to identify similar functional networks in both the control group and the subject, effectively eliminating the necessity to perform ICA separately on each subject and allowing differentiation of the same functional networks with different activation patterns. While there have been some concerns that the identification of aggregate functional networks using a single dataset of multiple groups may bias the results, a simulation study (Schmithorst and Holland, 2004) showed that GICA can identify a functional network that is present in only 10–15% of the total population. Another advantage of GICA is that the method is model-free and does not make any assumptions about the nature of BNC.

A novel finding of this study is the increased connectivity between sensorimotor and visual networks. The patient regained only 10% of sensory function in the lower body and relies heavily on vision to guide his movements. In light of this clinical presentation, our observation suggests plastic changes in the sensorimotor and visual networks of the patient. Studies on stroke patients have shown that visual guidance of movement contributes to the recovery of hand motor function (Kalra et al., 1997; Seitz et al., 1999), while lack of it may be detrimental for the recovery of sensorimotor function (Kalra et al., 1997). The increased functional connectivity between the sensorimotor and visual networks suggests that deprivation of sensory input that resulted from the SCI may have led to compensatory functional changes in the patient's brain. Another possibility is that the patient had increased connectivity between the two networks prior to the injury and this contributed to his recovery.

We also observed increased connectivity of the left executive network to other brain networks. The executive network (Seeley et al., 2007) links dorsolateral frontal and parietal cortex and is a task positive network that is related to working memory and control processes (Dosenbach et al., 2007; Stevens, 2009). The asymmetrical nature of the increased connectivity (left more than right) may be related to the asymmetric recovery of motor and sensory function of the patient. It should be noted that the magnitude of the asymmetry of the motor and sensory function is not fully appreciated through the AIS score, which is well-suited to describe the neurological level of the injury but ill-suited to describe the degree of functional loss.

The study presented here is a case report; therefore we are limited in our ability to draw statistically significant conclusions—for spatial differences of ICs as well as changes in BNC. This is because we are comparing single run data from multiple controls, subject to an inter-subject variability, to multiple run data from the subject, subject to an intra-subject variability. A larger cohort of SCI population is necessary in order to draw statistically significant conclusion about the changes in ICs and BNC of SCI patients. Nevertheless, the study showed good agreement between the rs-fMRI results and the clinical presentation of the subject and presents the possibility that rs-fMRI may aid in better SCI diagnosis and prognosis, as well as better monitoring and optimizing therapies for SCI subjects.

## Concluding remarks

In conclusion, the current study provides a detailed analysis of structural and functional changes in the central nervous system of a patient with chronic SCI who underwent a remarkable neurological recovery that continued for 17 years. To the best of our knowledge, this is the first study which used rs-fMRI to investigate cortical functional changes in SCI patients, alongside the investigation of structural changes using spinal cord imaging. Furthermore, the study has theoretical significance because it may identify variables—i.e., sufficient structural integrity for proper signal conduction in the spinal cord and compensatory plastic changes in the brain—that are necessary for neurological and functional improvements. Additionally, this work shows the potential of rs-fMRI to evaluate the extent of cortical plasticity required for functional recovery in patients whose neurological deficits prohibit completion of externally cued motor or sensory tasks.

### Conflict of interest statement

Dr. Pekar serves as Manager of the F. M. Kirby Research Center for Functional Brain Imaging, which receives research support from Philips HealthCare, which manufactures the MRI scanners used in this study. Dr. van Zijl has grant funding from Philips Healthcare. In addition, Dr. van Zijl is the inventor of technology that is licensed to Philips and is a paid lecturer for Philips. This arrangement has been approved by Johns Hopkins University in accordance with its conflict of interest policies. The other authors declare that the research was conducted in the absence of any commercial or financial relationships that could be construed as a potential conflict of interest.
